# Implicit motives predict affective responses to emotional expressions

**DOI:** 10.3389/fpsyg.2013.00985

**Published:** 2013-12-26

**Authors:** Andreas G. Rösch, Steven J. Stanton, Oliver C. Schultheiss

**Affiliations:** ^1^Department of Psychology and Sport Sciences, Friedrich-Alexander UniversityErlangen, Germany; ^2^Department of Management and Marketing, Oakland UniversityRochester, MI, USA

**Keywords:** inhibited power motive, implicit motives, activity inhibition, affect, emotion, arousal, valence

## Abstract

We explored the influence of implicit motives and activity inhibition (AI) on subjectively experienced affect in response to the presentation of six different facial expressions of emotion (FEEs; anger, disgust, fear, happiness, sadness, and surprise) and neutral faces from the NimStim set of facial expressions (Tottenham et al., 2009). Implicit motives and AI were assessed using a Picture Story Exercise (PSE) (Schultheiss et al., 2009b). Ratings of subjectively experienced affect (arousal and valence) were assessed using Self-Assessment Manikins (SAM) (Bradley and Lang, 1994) in a sample of 84 participants. We found that people with either a strong implicit power or achievement motive experienced stronger arousal, while people with a strong affiliation motive experienced less arousal and less pleasurable affect across emotions. Additionally, we obtained significant power motive × AI interactions for arousal ratings in response to FEEs and neutral faces. Participants with a strong power motive and weak AI experienced stronger arousal after the presentation of neutral faces but no additional increase in arousal after the presentation of FEEs. Participants with a strong power motive and strong AI (inhibited power motive) did not feel aroused by neutral faces. However, their arousal increased in response to all FEEs with the exception of happy faces, for which their subjective arousal decreased. These differentiated reaction patterns of individuals with an inhibited power motive suggest that they engage in a more socially adaptive manner of responding to different FEEs. Our findings extend established links between implicit motives and affective processes found at the procedural level to declarative reactions to FEEs. Implications are discussed with respect to dual-process models of motivation and research in motive congruence.

## Introduction

This paper investigates two questions. Firstly, it examines whether implicit motives (power, affiliation, and achievement motives) predict subjective affective reactions to other people's facial expressions of emotion (FEEs). Secondly, it explores whether the trait of activity inhibition (AI) moderates a potential relationship between the implicit power motive and affective responses.

Implicit motives are unconscious motivational dispositions (needs) that energize the attainment of motive-specific incentives, or the avoidance of motive-specific disincentives, by charging them with affect (Schultheiss, 2008). Currently, the “Big Three” implicit motives are the focus of most research (Schultheiss and Brunstein, 2010). For people with a strong need for power (nPower), having impact on others represents an incentive, while losing impact or being in a submissive position represents a disincentive. For people with a strong need for affiliation (nAffiliation), being in a close harmonious relationship represents an incentive, while separation or the loss of a relationship represents a disincentive. And finally, for people with a strong need for achievement (nAchievement) the autonomous mastery of challenging tasks represents an incentive, while failure in reaching a standard of excellence on one's own represents a disincentive, respectively (Atkinson, 1957; Schultheiss, 2008). Implicit motives are assessed with the Picture Story Exercise (PSE; McClelland et al., 1989), a derivative of Morgan's and Murray's (1935) Thematic Apperception Test, and do not correlate with the corresponding explicit questionnaire measures of motivation (McClelland et al., 1989; Spangler, 1992). Implicit motives are assumed to be part of brain circuits that have evolved earlier in evolution (Rolls, 1999; Schultheiss, 2013) and to respond preferentially to nonverbal stimuli, like FEEs, which are perceived and experienced directly, as compared to verbal stimuli, like spoken or written words, which represent symbolized knowledge that does not directly relate to subjective perception and experience. This distinction is in accordance with two-systems models of information processing (Schultheiss, 2001), which assume that verbal-symbolic stimuli trigger more conscious information processing, while experiential stimuli trigger more unconscious information processing, along with according modes of behavior.

For implicit motives, preferential processing of nonverbal-experiential stimuli has been demonstrated for various emotional and affective phenomena. Using a dot probe task (Mogg and Bradley, 1999), Schultheiss and Hale (2007) found that attentional orienting toward or away from FEEs depends on both perceivers' motive strength (nPower and nAffiliation) and the incentive salience of FEEs for a given motive. Wang et al. (2011) corroborated this finding by showing that the salience of FEEs depends on participants' motive strength (nPower), as assessed with motive-dependent differences in event-related potentials in a Stroop task with differently colored faces. Further evidence for the role of implicit motives in affective processes comes from studies in which procedural learning (e.g., of visuomotor sequences) was reinforced by FEEs, again depending on their incentive salience for a motive, as well as by social success and failure, e.g., in a dominance contest (reviewed in Schultheiss and Schiepe-Tiska, 2013). Finally, implicit motives have also been shown to determine subjective ratings of affect, such as emotional well-being in everyday life (Brunstein, 2010). Motive effects on emotional processes have been most consistently documented for nPower, while effects for nAffiliation and nAchievement remain to be clarified [for an overview see Stanton et al. (2010)]. Taken together, the studies reported above indicate that nonverbal stimuli, especially FEEs, convey information about the incentive salience of situational stimuli, which in turn determines a wide spectrum of affective reactions that depend on a person's implicit motives.

A frequent moderator of motive effects on behavior is AI, which is measured as the frequency of negations in spoken/written text (cf. Langens, 2010). As an example, the sentence “He did not study his opponent's tactics before the boxing match and therefore was not able to beat him,” written about the PSE image “boxer,” would be scored twice for AI. The validity of the AI construct has been demonstrated by its predictive power for a wide spectrum of outcome measures—from biological reactions like systolic blood pressure in response to a performance test (Fontana et al., 1987) to the overall economic performance or perceived greatness of political leaders (Spangler and House, 1991). In early conceptions, AI was assumed to represent a linguistic marker of an individual's mere ability to inhibit emotional impulses and behavior (e.g., McClelland and Boyatzis, 1982). In more modern conceptions, AI is understood as a propensity to engage emotion-processing functions of the right hemisphere, which are supposed to facilitate the flexible adjustment of behavior to challenging circumstances in social interactions (Schultheiss et al., 2009a). The right hemisphere also plays a key role in the encoding and decoding of nonverbal signals of emotion such as FEEs (e.g., Adolphs, 2002), which might be the mediating process for AI influences on various social outcomes. Thus, a person who is confronted with an ambiguous FEE that could either be interpreted as a neutral or hostile FEE, for instance, should better be able to thoroughly process the meaning of the stimulus and hence to react more adequately in terms of subjective affective reactions as well as actual behavior if he or she is high in AI, but not if he or she is low on this variable (Schultheiss et al., 2009a). Next to these AI main effects, AI influences can often be found in interaction with implicit motives. Jointly high levels of AI and nPower, the so called inhibited power motive, appear to be particularly predictive of physiology and behavior. The inhibited power motive has been linked to a more nonverbally expressive and persuasive communication style (Schultheiss and Brunstein, 2002), to more social and economic success (presumably because individuals with an inhibited power motive know how to read other people's nonverbal signals; cf. McClelland and Boyatzis, 1982), and to higher levels of sympathetic arousal (McClelland et al., 1980). Together, these findings indicate that high levels of AI let people high in nPower express their need for influence in a socially adaptive manner of affective-emotional behaviors and responses and thus to make these people navigate the social environment more successfully.

To sum up, previous research suggests that implicit motives, as well as the inhibited power motive, influence both the expression of one's own affect and subjective reactions to someone else's FEEs, along with associated physiological arousal. So far, however, motive-dependent responses to FEEs have only been reported for procedural measures of affective responses to emotion-eliciting stimuli, but not for declarative measures, like subjective affect ratings. Understanding if and when implicit motives and AI are reflected in subjective affective responses may help make people aware of their implicit motives and use this awareness for the selection and pursuit of motive-congruent goals.

In our study, we start to fill this gap by exploring effects of implicit motives in general and the inhibited power motive in particular on a declarative measure of affect in response to FEEs. We presented participants with six FEEs from the NimStim set (Tottenham et al., 2009), recorded their subjectively experienced arousal and valence in reaction to the presentation of these FEEs using the Self-Assessment Manikin (SAM; Bradley and Lang, 1994), and related individual differences in these ratings to participants' implicit motives and AI.

As previous research has shown, FEEs are an especially salient class of (dis-)incentives for nPower (Stanton et al., 2010). For arousal ratings, we therefore assumed that nPower is associated with pronounced affect ratings across all emotions. For valence ratings, however, we assumed no straightforward main effect, as nPower might amplify valence ratings in reaction to different FEEs into different directions—e.g., more positive reactions to FEEs that represent incentives and more negative reactions to FEEs that represent disincentives for that motive. As effects of nAffiliation and nAchievement remain to be clarified in the context of affective and emotional processes, we investigated their effects on affect ratings in an explorative way. Additionally, we explored the role of AI as a potential moderator of relationships between nPower and affect ratings.

## Materials and methods

### Participants

Ninety-five students enrolled at the University of Michigan, Ann Arbor, were tested (no Psychology majors) in a study approved by the Institutional Review Board of the University of Michigan. Nine participants with missing PSE data (due to technical problems), and two participants who did not follow the instructions (revealed by a lack of variance in their affect ratings) were excluded from analyses. The remaining 84 participants (59 women) were 19.65 years old on average (*SD* = 1.60).

### Design

Participants' implicit motives were measured between subjects. FEEs (anger, disgust, fear, happiness, sadness, surprise, and neutral expressions), face gender (female, male), and face race (African-American, Caucasian) were varied within subjects. Arousal and valence ratings in response to the FEEs were the dependent variables.

### Procedure

Participants first gave their informed consent. Then their implicit motives were assessed. After working on unrelated tasks (a short Implicit Association Task, two questionnaires, and a gender rating task), participants completed the affect ratings. Finally, participants provided demographic information, were debriefed and paid $30. Stimuli, instructions, and materials were presented via PCs. Responses were recorded with standard keyboards and mice.

### Measures

#### Implicit motives and activity inhibition

Implicit motives were assessed with the PSE, using the stimuli and instructions described in Schultheiss et al. (2009b). Participants wrote imaginative stories about eight pictures that were subsequently coded for motivational imagery (nPower, nAffiliation, and nAchievement) by an expert coder using Winter's (1994) manual. The scorer had previously exceeded the 85% interrater agreement criterion on calibration materials as a measure for scoring reliability and was blind to the research hypotheses. AI was determined per word-count software as the frequency of the negation “not” in its written-out and contracted variants in participants' PSE stories (see Schultheiss et al., 2009a). On average, participants wrote 919 (*SD* = 275) words, containing 4.74 (*SD* = 2.88) power, 5.64 (*SD* = 3.11) affiliation, 7.04 (*SD* = 2.91) achievement images, and 8.33 (*SD* = 6.33) AI scores summed across stories. Scores for test-retest reliability of motive scores derived from PSE stories are reported in Schultheiss and Pang (2007) and fall in the range from *r*_*tt*_ = 0.71 (1 day interval) to *r*_*tt*_ = 0.25 (10 years interval). Implicit motive and AI scores correlated with protocol length (all *p*< 0.072) and differed from normal distributions. Therefore, we followed the recommendations by Smith et al. (1992) and used square root transformations [sqr (1 + raw score)] to establish normality, corrected the transformed scores for protocol length using regression, and converted the residuals to z-scores. In so doing, we ensured that implicit motives scores follow a normal distribution and are not just a by-product of mere word count (people who write longer stories in the PSE would otherwise have higher implicit motives scores partly as a function of their higher verbal fluency). These z-scores were used for all analyses.

#### Affect ratings

We assessed participants' affective reactions to FEEs using the SAM scales (Bradley and Lang, 1994) for subjectively experienced arousal and valence. Participants were instructed to indicate, how the pictures (of facial expressions) make them feel using the two rating scales. For the arousal ratings, participants had to indicate whether a picture makes them feel anxious, calm, or somewhere in between. For the valence ratings, participants had to indicate whether a picture makes them feel sad, happy, or somewhere in between. Both scales ranged from 1 (no arousal/most sad) to 5 (highest arousal/most happy). All 96 stimuli were presented twice and participants rated their reaction to them first in terms of arousal and then in terms of valence, without time limits for responding. Internal consistencies for the different FEEs categories ranged from α = 0.92 to α = 0.95 (median α = 0.94) for arousal ratings and from α = 0.77 to α = 0.93 (median α = 0. 91) for valence ratings. Stimuli were randomly presented one at a time, with the instruction and the rating scale below the stimulus.

We used FEEs and neutral expressions from the NimStim set (Tottenham et al., 2009) as stimuli and chose pictures from the three persons with the highest prototypicality ratings of each of the following stimulus face categories: African-American women (#12, 13, 14), Caucasian women (#7, 9, 10), African-American men (#22, 27, 36), and Caucasian men (#40, 41, 43). From each of these twelve stimulus persons we used six FEEs (anger, disgust, fear, happiness, sadness, and surprise) and one neutral expression. Open-mouth versions were selected throughout, except for sad and neutral expressions. Faces were cropped so that each was visible from cheekbone to cheekbone and hairline to chin. Picture portions below the jawline were blackened and faces' heights were resized to 19.5 cm (width varied due to posers' physiognomy). To control for general response tendencies to pictorial cues, we additionally included a set of twelve neutral stimuli.

### Analyses

To maximize test power and to accommodate the continuous motive variables, we ran (repeated-measures) regression analyses in SYSTAT 13. Only the within-subject factor “FEE” was considered for analyses, but not the factors gender and race, as they were merely introduced to obtain a representative set of stimulus images.

## Results

### Descriptive statistics

Descriptive statistics of affect ratings in response to facial expressions as well as their intercorrelations with implicit motive and AI scores are summarized in Table 1. Significant differences for arousal and valence ratings between FEEs emerged [all *F*_(5, 415)_> 197.15; all *p* < 0.001]. *Post-hoc t*-tests with *p* set at 0.05 revealed an anger > disgust = fear > surprise = sadness > happiness relation for arousal ratings and an anger < disgust = fear = sadness < surprise < happiness relation for valence ratings. For neutral expressions, arousal ratings differed from the neutral offset, defined as the scale minimum [*t*_(83)_ = 12.78, *p* < 0.001] and valence ratings tended to be lower than the neutral midpoint of the scale [*t*_(83)_ = −1.74, *p* = 0.085].

**Table 1:** **Means, *SD*s, and raw correlations of implicit motives (z-scores), activity inhibition (z-score) and affect ratings in response to the presentation of six facial expressions of emotion and neutral faces (arousal ratings below diagonal; valence ratings above diagonal)**.

**Variable**	**Mean (*SD*)**	**1**	**2**	**3**	**4**	**5**	**6**	**7**	**8**	**9**	**10**	**11**
Mean (*SD*)		0.00 (1.00)	0.00 (1.00)	0.00 (1.00)	0.00 (1.00)	1.99 (0.69)	2.13 (0.67)	2.27 (0.62)	4.15 (0.70)	2.27 (0.51)	2.89 (0.56)	2.93 (0.36)
1. nAchievement	0.00 (1.00)	—	0.35^**^	0.05	−0.00	−0.16	−0.17	−0.25^*^	−0.10	−0.17	−0.07	−0.01
2. nAffiliation	0.00 (1.00)	0.35^**^	—	0.09	0.01	−0.20^#^	−0.19^#^	−0.20^#^	0.08	−0.14	−0.11	−0.16
3. nPower	0.00 (1.00)	0.05	0.09	—	−0.16	−0.15	−0.18^#^	−0.10	0.12	−0.11	−0.16	−0.00
4. AI	0.00 (1.00)	−0.00	0.01	−0.16	—	−0.14	−0.11	−0.21^#^	0.09	−0.05	−0.04	0.06
5. Anger	3.84 (0.71)	0.17	0.03	0.27^*^	0.10	−0.56^***^	−0.59^***^	−0.54^***^	0.16	−0.42^***^	−0.29^**^	−0.09
6. Disgust	3.55 (0.80)	0.20^#^	−0.09	0.26^*^	0.11	−0.45^***^	−0.60^***^	−0.56^***^	0.05	−0.36^***^	−0.38^***^	−0.11
7. Fear	3.48 (0.74)	0.21^#^	0.01	0.20^#^	0.15	−0.45^***^	−0.55^***^	−0.58^***^	0.03	−0.32^**^	−0.35^**^	−0.05
8. Happiness	1.67 (0.71)	0.21^#^	0.03	0.02	0.00	−0.02	−0.10	−0.15	−0.45^***^	0.09	−0.36^***^	0.02
9. Sadness	2.76 (0.79)	0.23^*^	−0.08	0.22^*^	0.03	−0.40^***^	−0.46^***^	−0.46^***^	−0.11	−0.32^**^	−0.38^***^	−0.14
10. Surprise	2.84 (0.73)	0.27^*^	0.04	0.15	0.15	−0.41^***^	−0.47^***^	−0.55^***^	−0.10	−0.32^**^	−0.53^***^	−0.18
11. Neutral	2.02 (0.74)	0.12	−0.02	0.15	−0.03	−0.19^#^	−0.24^*^	−0.23^*^	−0.24^*^	−0.05	−0.29^**^	−0.20^#^

# p < 0.100;

* p < 0.050;

** p < 0.010;

*** *p < 0.001*.

Averaged across FEEs, arousal and valence ratings correlated negatively (*r* = −0.64, *p* < 0.001), with greater variance in arousal than valence ratings [*F*_(83, 83)_ = 2.35, *p* < 0.001]. nAchievement and nAffiliation correlated positively (*r* = 0.35, *p* = 0.001).

### Motivational influences on affect ratings

#### Motive influences on affect ratings in response to neutral faces

To rule out associations between motivational predictors and affect ratings that are caused simply by viewing a human face, we first explored whether implicit motives and AI were associated with arousal and valence ratings in response to neutral faces. For this purpose, we ran two separate regression analyses for each affect dimension (a total of four regressions). Regression analyses included either the individual motive scores and AI simultaneously, or alternatively nPower, AI, and their interaction term as predictors. In light of the variance overlap between arousal and valence ratings, we controlled for valence ratings in response to the neutral faces when testing for arousal ratings and vice versa in all regression analyses.

An analysis with arousal ratings as dependent variable revealed a significant nPower × AI interaction effect (see Table 2) ^1^. This interaction effect differed only marginally when we repeated the analysis using an AI median-split predictor (*B* = −0.275, *SE* = 0.160, *t* = −1.72, *p* = 0.090). *Post-hoc* analyses using an AI median split showed that nPower was associated with higher arousal ratings of neutral expressions in low-AI individuals (*t* = 2.23, *p* = 0.031, semipartial *r* = 0.34), but not in high-AI individuals (*t* = −0.16, *p* = 0.874, semipartial *r* = −0.03).

**Table 2:** **Test statistics of the regression analysis predicting arousal ratings in response to neutral faces from the interaction of nPower and AI**.

**Variable**		***B***	***SE***	***t***	***p***
Constant		3.188	0.650	4.90	<0.001
Valence (Neutral)		−0.406	0.220	−1.85	0.069
nPower		0.118	0.079	1.50	0.138
AI		−0.014	0.080	−0.18	0.859
nPower × AI		−0.178	0.086	−2.08	0.041
*R*^2^	0.11				
*F*_(4, 79)_	2.46^#^				

*Valence (Neutral), Valence rating in response to neutral faces; nPower, implicit power motive; AI, activity inhibition*.

# *p = 0.052*.

Neither the effects of individual implicit motives or AI on affect measures, nor the nPower × AI interaction effect on valence ratings became significant (all *t* < |1.44|, all *p* >0.154)^2^. (For regression analyses on valence, ratings of three outliers with studentized residuals up to 3.82 had to be removed from analysis.) Taken together, these findings suggest that AI and nPower jointly bias ratings on non-emotional faces to a substantial degree. All further analyses on the influence of implicit motives and AI should therefore control for affect ratings in response to neutral faces.

#### Motive influences on affect ratings in response to facial expressions of emotion

Due to the results for neutral faces and also due to the variance overlap between valence and arousal ratings as well as between implicit motives, in subsequent analyses we simultaneously included (a) all implicit motive and AI scores (or alternatively nPower, AI, and their interaction term), (b) same-scale ratings for neutral expressions, and (c) other-scale ratings of FEEs as predictors in the regression analyses predicting affect ratings in response to FEE (e.g., analyses on arousal ratings included average arousal ratings for neutral expressions and average valence ratings for FEEs as predictors).We thus ensured that implicit motive and AI (interaction) effects on arousal or valence ratings were not influenced by variance overlap between motive scores, overall response bias (ratings on neutral expressions), and the high variance overlap between arousal and valence ratings. *Post-hoc* analyses accordingly controlled for these factors as well. As for the analyses on neutral faces, this resulted again in a total of four (repeated-measures) regression analyses—two for each affect dimension.

A first regression analysis on arousal ratings with individual motive scores and AI as predictors indicated that nPower was marginally associated with higher arousal ratings (semipartial *r* = 0.18), nAffiliation was significantly associated with lower arousal ratings (semipartial *r* = −0.22), and nAchievement was significantly associated with higher arousal ratings (semipartial *r* = 0.21) of FEEs (see Table 3 for test statistics)^3^.

**Table 3:** **Test statistics of the regression analyses predicting arousal ratings in response to facial expressions of emotion**.

**Variable**		***B***	***SE***	***t***	***p***
Constant		4.087	0.354	11.55	<0.001
Arousal (Neutral)		0.385	0.059	6.48	<0.001
Valence (Emotion)		−0.704	0.113	−6.25	<0.001
nAchievement		0.090	0.044	2.03	0.045
nAffiliation		−0.097	0.044	−2.20	0.031
nPower		0.069	0.042	1.64	0.105
AI		0.056	0.042	1.34	0.184
*R*^2^_*adjusted*_	0.64				
*F*_(6, 77)_	25.54^***^				

*Arousal (Neutral), arousal rating in response to neutral faces; Valence (Emotion), average valence rating in response to facial expressions of emotion; nAchievement, implicit achievement motive; nAffiliation, implicit affiliation motive; nPower, implicit power motive; AI, activity inhibition*.

*** *p < 0.001*.

**Figure 1:**
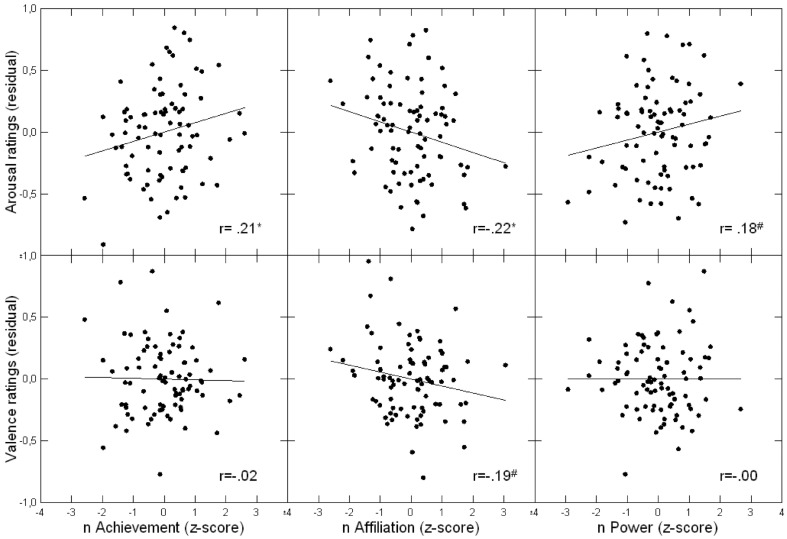
**Scatterplots of associations between implicit motives and affect ratings (averaged across emotions)**. ^*^p < 0.05; ^#^*p* < 0.10.

A second regression analysis on valence ratings with individual motive scores and AI as predictors indicated that nAffiliation was marginally associated with lower valence ratings (semipartial *r* = −0.19), while effects of nPower and nAchievement did not reach significance (see Table 4 for test statistics)^4^. Motive main effects on both affect dimensions are illustrated in Figure 1.

**Table 4:** **Test statistics of the regression analyses predicting valence ratings in response to facial expressions of emotion**.

**Variable**		***B***	***SE***	***t***	***p***
Constant		3.273	0.356	9.19	<0.001
Valence (Neutral)		0.188	0.096	1.97	0.053
Arousal (Emotion)		−0.399	0.059	−6.78	<0.001
nAchievement		−0.008	0.037	−0.21	0.832
nAffiliation		−0.068	0.036	−1.88	0.064
nPower		−0.001	0.035	−0.02	0.985
AI		−0.024	0.034	−0.72	0.477
*R*^2^_adjusted_	0.44				
*F*_(6, 77)_	11.90^***^				

*Valence (Neutral), valence rating in response to neutral faces; Arousal (Emotion), average arousal rating in response to facial expressions of emotion; nAchievement, implicit achievement motive; nAffiliation, implicit affiliation motive; nPower, implicit power motive; AI, activity inhibition*.

*** *p < 0.001*.

**Figure 2:**
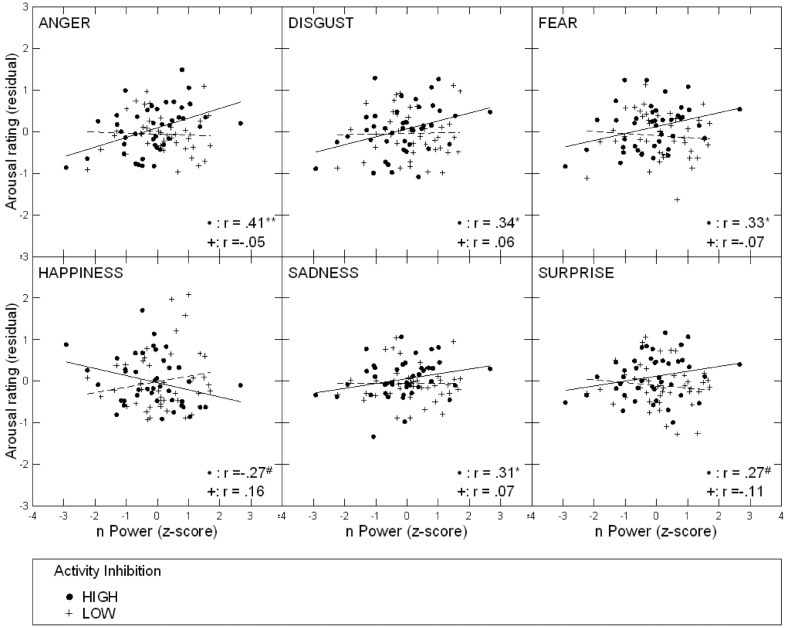
**Scatterplots of associations between nPower and arousal ratings for different emotions, depending on participants' AI level; Solid circles and lines: people high in AI; crosses and dashed lines: people low in AI**. ^**^p < 0.01; ^*^p < 0.05; ^#^*p* < 0.10.

A third repeated-measures regression analysis on arousal ratings revealed a significant nPower × AI × emotion interaction effect [*F*_(5, 390)_ = 3.46, *p* = 0.005, η^2^ = 0.036]^5^. This interaction effect differed only marginally when we repeated the analysis using an AI median-split predictor [*F*_(5, 390)_ = 4.74, *p* < 0.001, η^2^ = 0.048]. Follow-up regressions resulted in a significant nPower × emotion interaction for high-AI (above median) individuals [*F*_(5, 190)_ = 4.77, *p* < 0.001, η^2^ = 0.083], but not for low-AI (below median) individuals [*F*_(5, 190)_ = 0.94, *p* = 0.454, η^2^ = 0.022]. After plotting and inspecting the interaction separately for all six FEEs (see Figure 2), we ran a *post-hoc* comparison of regression slopes of the FEE of happiness against the average regression slope of all other FEEs (i.e., comparing positively and negatively valenced FEEs). A follow-up repeated-measures regression with high-AI individuals revealed a significant nPower × emotion interaction [*F*_(1, 38)_ = 8.20, *p* = 0.007, η^2^ = 0.140]. Contrasting these FEEs best summarizes the original interaction effect. In high-AI participants, arousal ratings for negatively valenced FEEs increased with nPower (*B* = 0.18, *SE* = 0.07, semipartial *r* = 0.39, *p* = 0.011), while they tended to decrease for happiness (*B* = −0.16, *SE* = 0.09, semipartial *r* = −0.27, *p* = 0.089).

A fourth repeated-measures regression analysis predicting valence ratings could not find a similar nPower × AI × emotion interaction effect [*F*_(5, 385)_ = 0.66, *p* = 0.653, η^2^ = 0.007]^6^. (One outlier with a studentized residual of 3.43 had to be removed from this analysis.)

## Discussion

In this study, we explored effects of implicit motives and AI on ratings of subjectively experienced affect in response to six FEEs. Our results provide evidence for associations of all three implicit motives with arousal ratings, and of nAffiliation with valence ratings. People high in nAchievement or nPower experience the presentation of other people's FEEs as overall more arousing compared to people low in either of these motives. However, these motives do not influence how pleasant people experience the presentation of these FEEs. People high in nAffiliation, in turn, experience the presentation of other people's FEEs as overall less arousing and more unpleasant compared to people low in this motive. Additionally, for the combination of high levels of AI and nPower, the inhibited power motive, a pattern of emotion-specific modulation of arousal ratings in response to neutral faces and FEEs emerged. In low-AI individuals, nPower was associated with stronger feelings of arousal in response to the presentation of neutral faces, but no additional increase of subjectively experienced arousal in response to FEEs, relative to neutral faces. In contrast, in high-AI individuals, nPower was not associated with arousal ratings in response to neutral expressions, but with weaker subjective arousal in response to the presentation of happy faces and with stronger subjective arousal in response to the presentation of all other FEEs.

Considering the association between nPower and arousal ratings, results confirm the notion that implicit motives amplify affective responses to motivational incentives (Atkinson, 1957) and previous research underscores that FEEs are motive-specific (dis-)incentives that energize behavior. The motive-dependent accentuation of overall arousal ratings might therefore reflect a greater sensitivity for and reactivity to FEEs that represent relevant feedback signals with learned meaning for the pursuit of one's motive-dependent goals in social interactions (Schultheiss, 2008).

Moreover, the additional nPower × AI × emotion interaction effect on arousal ratings differentiates the nPower main effect and shows that individuals with an inhibited power motive are able to better differentiate explicitly between FEEs and experience different arousal intensities in response to FEEs with differing signal value. This is remarkable, as typically the inhibited power motive is associated with socially effective behavior and success, whereas uninhibited power motivation tends to be associated with a more impulsive, exploitative and hostile interaction style (Schultheiss, 2008). We speculate that differential responses by individuals with and without inhibited power motive to interaction partners' FEEs may be partly responsible for these divergent behavioral outcomes. Individuals with an inhibited power motive may perceive happy FEEs as an indication of their own positive emotional impact on another person, which is reinforcing for them, while they correctly perceive negative FEEs as indications of a lack in their goal progress or as a threat to their own dominance. In the long run, more differentiated responses of individuals with an inhibited power motive to specific FEEs in others might foster positive and successful social interactions. In contrast, individuals with an uninhibited power motive simply seem to be aroused by faces in general and not by specific FEEs. This lack of response differentiation could hamper adequate social interactions. This result might be important in a broader theoretical context of social interactions, e.g., for different behavioral modes of obtaining higher status in social hierarchies (e.g., Henrich and Gil-White, 2001; Cheng et al., 2010, 2013), but we avoid far reaching interpretations, as the nPower x AI interaction effects needs to be replicated first.

As a limitation of our study, missing effects of nPower or the inhibited power motive on valence ratings might be attributed to the lower variance in these ratings or the notion that valence ratings of FEEs is highly over-learned or over-determined by culture (Russell, 1994) and therefore not sensitive to motive-dependent modulation. Moreover, nAchievement predicted arousal ratings positively and nAffiliation predicted both affect dimensions negatively. These findings show that the affect amplifier property of implicit motives generalizes beyond the domain of power to all motives under investigation. However, we avoid interpreting these preliminary findings, as a replication of the main effects of nAchievement and nAffiliation seems mandatory before doing so. However, it is worth noting that the effects for nAffiliation could only be demonstrated when we explored the specific variance in arousal and valence ratings, which cannot be attributed to the presentation of faces *per se* (ensured by controlling for the corresponding ratings for neutral faces) or to the large amount of shared variance between the two affect measures (ensured by controlling for the second affect dimension in each analysis). Replication studies should take this approach into account.

Another limitation of our study comes from the affect rating task itself. Participants were presented with a high number of different FEEs in quick succession. This raises the question if the affect ratings in our study can be compared to naturally elicited affect. In our opinion, affective reactions from our participants do only differ from natural affect in terms of intensity, as changes in affect are rapidly occurring reactions under natural circumstances as well (see Oatley et al., 2006). Additionally, recent fMRI studies of affective responses to emotion-eliciting events demonstrate that brain activations, subjective feelings, as well as accompanied visceral changes occur rapidly upon elicitation (Wager et al., 2009). Nevertheless, we acknowledge that the results reported here have to be replicated under more naturalistic conditions.

Taken together, our findings are consistent with recent work on implicit motives and FEEs (see Stanton et al., 2010). They confirm that incentive effects of perceived FEEs, previously assessed with procedural measures, extend to declarative measures of subjectively experienced affect in reaction to FEEs. If individuals aim to react appropriately to different FEEs, depending on their relevance for a given implicit motive, FEEs need to be appraised accordingly (Frijda, 2007). As implicit motives operate outside of conscious awareness, this points to the notion that appraisal of FEEs may proceed partly at an unconscious level (cf. Moors, 2010). Additionally, our results are important as they indicate that implicit motives unfold their influence not only at the level of unconscious and procedural processes, which are inaccessible for introspection, but also to some degree at the level of conscious affective processes. In the context of established two-process models of motivation (e.g., McClelland et al., 1989; Schultheiss, 2001), our results point to affect as a potential means of between-systems information exchange, making the output of the implicit system available to the explicit system. Focusing on or becoming aware of one's affective reaction in specific situations (e.g., observing other people's FEEs) could make the implicit-explicit barrier more permeable, as subjectively experienced affect can be understood as a valid indicator of progress toward one's implicit needs.

## Conclusion

This research has shown that affective responses to FEEs are influenced by the interplay between FEEs and perceivers' implicit motives and AI. Future studies should establish causality for these associations, extend our understanding of these associations by incorporating indirect measures of affect (e.g., skin conductance and heart rate), and compare these with results from declarative measures of affect.

## Author contributions

Andreas G. Rösch conducted all data analyses, prepared figures and tables as well as the manuscript. Parts of the results have been previously published in his dissertation thesis. Steven J. Stanton prepared and conducted the experiment at the University of Michigan and revised the manuscript. Oliver C. Schultheiss supervised all phases from preparation of the experiment to data analyses and further revised the manuscript.

### Conflict of interest statement

The authors declare that the research was conducted in the absence of any commercial or financial relationships that could be construed as a potential conflict of interest.

## Footnotes

^1^The nPower × AI interaction effect remained significant when we did not control for valence ratings of neutral faces (*B* = −0.177, *SE* = 0.087, *t* = −2.04, *p* = 0.044).

^2^None of these effects became significant when we did not control for arousal ratings in the prediction of valence ratings or vice versa (all *t* < |1.37|, all *p* > 0.174).

^3^When we did not control for valence ratings of FEEs, the overall regression analysis remained significant [*F*_(5, 78)_ = 15.35; *p* = 0.001, *R*^2^_*adjusted*_ = 0.46]. The main effects of nAchievement (*B* = 0.134, *SE* = 0.053, *t* = 2.50, *p* = 0.014, semipartial *r* = 0.25) and nPower (*B* = 0.099, *SE* = 0.051, *t* = 1.94, *p* = 0.056, semipartial *r* = 0.21) became stronger, while the effect of nAffiliation (*B* = −0.059, *SE* = 0.053, *t* = −1.10, *p* = 0.274, semipartial *r* = −0.12) was no longer significant. Additionally, a marginally significant main effect of AI (*B* = 0.097, *SE* = 0.050, *t* = 1.93, *p* = 0.057, semipartial *r* = 0.21) emerged. When we additionally did not control for arousal ratings of neutral faces the pattern of results stayed the same, however the main effect of AI could no longer be found.

^4^ When we did not control for arousal ratings of FEEs, the overall regression analysis remained significant [*F*_(5, 78)_ = 3.23; *p* < 0.011, *R*^2^_*adjusted*_ = 0.12]. The main effects of nAffiliation (*B* = −0.027, *SE* = 0.045, *t* = −0.60, *p* = 0.550, semipartial *r* = −0.06) was no longer significant, while the effect of nAchievement (*B* = −0.083, *SE* = 0.044, *t* = −1.88, *p* = 0.064, semipartial *r* = −0.19) emerged as a tendency. Main effects of nPower (*B* = −0.064, *SE* = 0.042, *t* = −1.51, *p* = 0.135, semipartial *r* = −0.17) or AI (*B* = −0.064, *SE* = 0.042, *t* = −1.53, *p* = 0.131, semipartial *r* = −0.17) could not be found. Additionally not controlling for valence ratings of neutral faces did not further change the pattern of results.

^5^The nPower × AI interaction effect on arousal ratings remained significant when we did not control for valence ratings of FEEs [*F*_(5, 395)_ = 4.30, *p* < 0.001, η^2^= 0.037]. Additionally not controlling for arousal ratings of neutral faces did not further change the pattern of results.

^6^When we did not control for arousal ratings of FEEs a significant nPower × AI × Emotion interaction effect emerged [*F*_(5, 395)_ = 3.00, *p* = 0.011, η^2^ = 0.031]. However, when we excluded one participant who was marked as outlier in the original analysis, this interaction effect disappered completely [*F*_(5, 390)_ = 0.77, *p* = 0.575, η^2^ = 0.008]. Additionally not controlling for arousal ratings of neutral faces did not further change the pattern of results.
